# Nonprehensile Manipulation of Parts on a Horizontal Circularly Oscillating Platform with Dynamic Dry Friction Control

**DOI:** 10.3390/s21165581

**Published:** 2021-08-19

**Authors:** Sigitas Kilikevičius, Kristina Liutkauskienė, Algimantas Fedaravičius

**Affiliations:** Department of Transport Engineering, Kaunas University of Technology, Studentų St. 56, 51424 Kaunas, Lithuania; kristina.liutkauskiene@ktu.lt (K.L.); algimantas.fedaravicius@ktu.lt (A.F.)

**Keywords:** nonprehensile manipulation, dry friction, vibration and control, oscillating platform, planar motion

## Abstract

This paper presents a novel method for nonprehensile manipulation of parts on a circularly oscillating platform when the effective coefficient of dry friction between the part and the platform is being dynamically controlled. Theoretical and experimental analyses have been performed to validate the proposed method and to determine the control parameters that define the characteristics of the part’s motion. A mathematical model of the manipulation process with dynamic dry friction control was developed and solved. The modeling showed that by changing the phase shift between the function for dynamic dry friction control and the function defining the circular motion of the platform, the part can be moved in any direction as the angle of displacement can be controlled in a full range from 0 to 2*π*. The nature of the trajectory and the mean displacement velocity of the part mainly depend on the width of the rectangular function for dynamic dry friction control. To verify the theoretical findings, an experimental setup was developed, and experiments of manipulation were carried out. The experimental results qualitatively confirmed the theoretical findings. The presented analysis enriches the classical theories of nonprehensile manipulation on oscillating platforms, and the presented findings are relevant for mechatronics, robotics, mechanics, electronics, medical, and other industries.

## 1. Introduction

Two-dimensional manipulation and transportation of various objects are commonly used in many fields such as research, various industries, and medicine. There are many processes in manufacturing where objects/parts require sorting, positioning, transportation, orientation, classification, and/or assembly. These tasks can be carried out using either prehensile or nonprehensile methods. Prehensile methods usually involve grasping by some sort of grippers. In the case of nonprehensile manipulation, the parts to be manipulated are subjected only to unilateral constraints, e.g., placing an object on a platform. Nonprehensile manipulation methods have a lot of advantages, such as when there is no involvement of gripping, picking up, or other type of force or form closure, the stress on the part is minimum, workspace can be large, and lower operational times are required. A major advantage of nonprehensile manipulation is that it can be achieved with relatively simpler technological equipment.

Parts on a horizontal platform can be moved by various means. For example, this can be performed by using parallel manipulators [1,2,3,4] by applying piezoelectric or electromagnetic actuated planar micromanipulators [5,6,7,8], by pushing with robot end-effectors [9,10,11], by transporting with devices which move together with the parts to be displaced (e.g., mobile robots) [12,13,14], by employing actuator arrays under a flexible surface [15], by applying acoustic manipulation techniques [16,17,18,19], etc.

Methods utilizing vibrations are very versatile as they can be applied to manipulate objects of various sizes (ranging from nano/micro scale to heavy parts). Additionally, these methods can be applied to manipulate high temperature parts at contaminated environments, and they are suitable for individual parts and bulk materials. Moreover, they do not require expensive and complex equipment. Therefore, these methods are being extensively studied in scientific literature and applied in industrial practice [20].

There were many attempts to employ flexural vibrations induced in vibrating plates for nonprehensile manipulation task as the position and orientation of the particle can be controlled depending on the node shapes of the plate induced by particular frequencies [21,22,23,24,25]. However, these methods are mostly suited for manipulating micro-scale particles.

Frictional forces play a major role in nonprehensile manipulation methods that employ vibrating platforms. To make a part move on an oscillating platform, an asymmetry must exist to ensure that net friction forces over one oscillation cycle do not cancel out. Frei et al. [26] demonstrated that friction forces can be employed to move parts along a horizontal platform by vibrating the platform with two degrees of freedom. The horizontal and vertical oscillations were excited to generate a system asymmetry causing a non-zero resultant net friction force. By placing some small actuator cells next to each other, individual trajectories can be imposed on multiple parts. However, it requires a large array of lots of individual actuator cells, and achievable trajectories are limited to the geometrical parameters of the cells. Umbanhowar et al. [27] studied the role of anisotropic friction of textured surfaces such as micro-machined silicon and fabrics and showed that the asymmetry due to direction-dependent surface friction properties can be used in conjunction with an asymmetrically oscillating platform to help design friction-induced velocity fields on the surface of the plate, which can be employed for manipulation tasks.

Mayyas [28] analyzed the stick-slip dynamics of a part moving on an oscillating platform, intended to be used for two-dimensional manipulation. A temporal system asymmetry (also called time-asymmetry, it occurs when the forward and reverse motion have different speeds) was applied, and the part’s motion forward was possible when the platform acceleration in the forward direction generated an inertial force, which was lower than the friction force, or the backward acceleration generated an inertial force, which was higher than the friction force. A vibrating platform actuated by one actuator via a mechanism comprised of an active joint and a passive viscoelastic joint was proposed for manipulation tasks by Higashimori et al. [29]. In this case, omnidirectional nonprehensile manipulation was implemented by controlling the orientation and shape of the spatially asymmetric vibrational orbit of the platform by changing the frequency and offset angle of the sinusoidal displacement input to the actuator. 

Reznik et al. [30] studied body motion on a vibrating platform by applying asymmetric excitation. The motion of parts along a straight-line trajectory was achieved through this temporal asymmetry. Reznik et al. [31] proposed a method of nonprehensile manipulation on a platform excited by several actuators in an asymmetric manner to create average frictional force fields. This asymmetric excitation allowed them to move several parts individually. Viswarupachari et al. [32] investigated the directional motion of a part over a flat horizontal rigid platform vibrating asymmetrically. The directional movement was feasible as their system exhibited both the spatial asymmetry and the temporal asymmetry. A nonprehensile manipulation method employing a horizontal oscillating platform was investigated by Liutkauskienė et al. [33]. The dynamic directionality necessary for the part’s motion was accomplished through the asymmetry created as different frequencies with a phase shift were excited for the x and y directions of the platform. This sort of excitation eliminates the spatial symmetry. It was observed that the part can be moved along a defined trajectory by controlling the frequencies and the phase shift. A vibrating trough with finlike asperities was employed for handling of granular materials by Chen et al. [18]. The directional movement of the material was caused by the force asymmetry generated by the finlike asperities.

Fedaravičius et al. [34] proposed a vibrational transportation method by creating a frictional system asymmetry. Parts were transported along a straight line on a platform subjected to horizontal harmonic oscillations while manipulating with the friction force by periodically exciting additional vibrations in the contact zone. Dunst et al. [35] proposed a method for handling of dry fine powders by employing coordinated manipulations of effective friction. A pipe subjected to axial low frequency vibrations was applied for powder handling. The dynamic manipulations with the effective friction force were carried out by periodically exciting radial high frequency vibrations of the tube. This technique demonstrated an increased flowability, decreased adhesion, and lower agglomeration degree.

The literature review showed that an asymmetry is a necessary condition to achieve the part’s motion on a periodically oscillating platform. Temporal, spatial, or force asymmetries are commonly used in nonprehensile manipulation systems that utilize vibrations. The novelty of this work is that a horizontal platform subjected to circular oscillating motion is employed for omnidirectional nonprehensile manipulation while other systems employ other types of oscillating motion, and a frictional asymmetry is created by dynamically controlling the effective dry friction between the part and the platform instead of temporal, spatial, or force asymmetries that are commonly used in other nonprehensile manipulation methods that utilize vibrations. The effective friction force can be reduced by exciting high frequency vibrations [36,37,38,39,40]. Therefore, the effective dry friction force can be dynamically reduced in a predefined manner in respect of the period of the circular motion of the platform by periodically exciting high frequency vibrations in the contact zone. The purpose of the presented analysis is to propose a method of nonprehensile manipulation employing dry friction control and to determine the parameters that define the characteristics of the part’s motion.

## 2. Theoretical Analysis

### 2.1. Dynamics of Manipulation Employing Dry Friction Control

**Figure 1:**
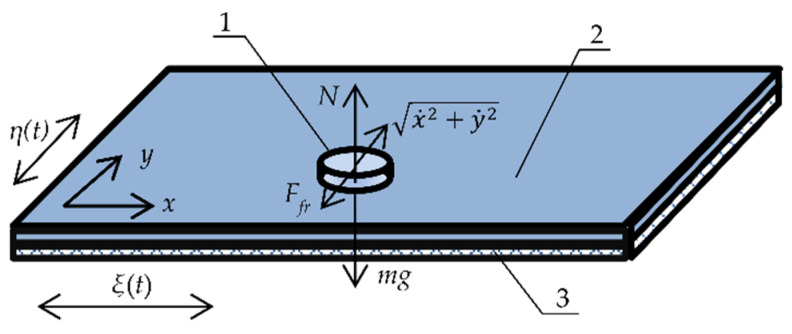
Dynamic model of nonprehensile manipulation on a circularly oscillating platform with dry friction control: (1) part; (2) platform; (3) piezoelectric actuator.

The dynamic model of manipulation of a part on a circularly oscillating platform using dry friction is shown in Figure 1.

The manipulation system consists of a horizontal platform excited in two perpendicular directions by harmonic excitation with a phase shift:

(1) $$\left\{ \begin{array}{l} \xi =\xi_{0}+A_{e}\cos\omega t,\\ \eta =\eta_{0}+A_{e}\cos\left(\omega t+\varepsilon \right), \end{array}\right.$$

where *t* is time, *A_e_* is the excitation amplitude (the radius of the circular motion), *ω* is the angular frequency of the circular excitation, and *ε* is the phase shift between the harmonic waves along the *ξ* and *η* axes (it is equal to *π*/2 for circular motion).

It is assumed that the part is flat, and the mass of the part is concentrated in the mass center. When the part is sliding in respect to the platform, the projections the acceleration of the mass center are written as follows:

(2) $$\left\{ \begin{array}{l} a_{\xi }=\ddot{x}+\ddot{\xi }=\ddot{x}-A_{e}\omega^{2}\cos\omega t,\\ a_{\eta }=\ddot{y}+\ddot{\eta }=\ddot{y}-A_{e}\omega^{2}\cos\left(\omega t+\varepsilon \right). \end{array}\right.$$

The part’s motion on the platform is influenced by the dry friction force *F_fr_* that acts opposite to the relative velocity expressed as $\sqrt{{\dot{x}}^{2}+{\dot{y}}^{2}}$. The projections of the dry friction force are written as follows:

(3) $$\left\{ \begin{array}{l} F_{fr\;\xi }=-\mu \left(\omega t\right)mg\cos\left(\sqrt{{\dot{x}}^{2}+{\dot{y}}^{2}},x\right)=-\mu \left(\omega t\right)mg\frac{\dot{x}}{\sqrt{{\dot{x}}^{2}+{\dot{y}}^{2}}},\\ F_{fr\;\eta }=-\mu \left(\omega t\right)mg\cos\left(\sqrt{{\dot{x}}^{2}+{\dot{y}}^{2}},y\right)=-\mu \left(\omega t\right)mg\frac{\dot{y}}{\sqrt{{\dot{x}}^{2}+{\dot{y}}^{2}}}. \end{array}\right.$$

Then, the following equilibrium can be written:

(4) $$\left\{ \begin{array}{l} ma_{\xi }=F_{fr\;\xi },\\ ma_{\eta }=F_{fr\;\eta }. \end{array}\right.$$

Inserting Equations (2) and (3) into Equation (4), the equations of motion of the part on the platform are written as follows:

(5) $$\left\{ \begin{array}{l} \ddot{x}+\mu (\omega t)g\frac{\dot{x}}{\sqrt{{\dot{x}}^{2}+{\dot{y}}^{2}}}=A_{e}\omega^{2}\cos\omega t,\\ \ddot{y}+\mu (\omega t)g\frac{\dot{y}}{\sqrt{{\dot{x}}^{2}+{\dot{y}}^{2}}}=A_{e}\omega^{2}\cos\left(\omega t+\varepsilon \right), \end{array}\right.$$

where *μ*(*ωt*) is the rectangular function for dynamic dry friction control. 

**Figure 2:**
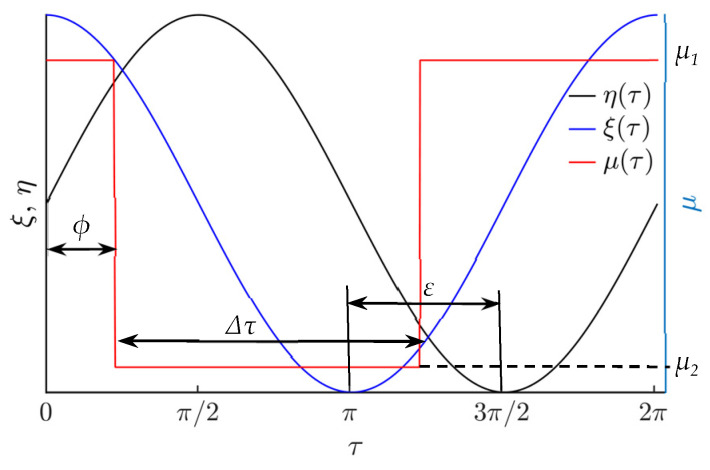
Principle of dry friction control in respect of the period of the function defining the circular motion of the platform.

The effective dry friction coefficient is being controlled in respect of the period of the function defining the circular motion of the platform by the following rectangular function:

(6) $$\mu (\omega t)=\left\{ \begin{array}{l} \mu_{2},\;when\;\phi +2\pi n<\omega t<\phi +\Delta \tau +2\pi n,\\ \mu_{1},\;for\;all\;other\;\omega t\;values, \end{array}\right.$$

where *n* = (0, 1, 2, …), *µ*_1_ is the nominal dry friction coefficient between the platform surface and the part, *µ*_2_ is the dynamically reduced effective dry friction coefficient, *ϕ* is the phase shift between the function for dynamic dry friction control and the function defining the circular motion of the platform, Δ*τ* is the width of the rectangular function for dynamic dry friction control (Figure 2).

The parameter Δ*τ* can also be viewed as a fraction of the period of the circular oscillations where the effective dry friction coefficient is reduced (becomes equal to *µ*_2_). The principle of dynamic dry friction control is presented in Figure 2. As it was discussed earlier, a dynamic reduction in the effective dry friction coefficient can be performed by exciting high frequency vibrations between the platform and the part.

### 2.2. Modeling Results

A numerical modeling of part manipulation was carried out. The modeling software was developed in the MATLAB (MathWorks, Natick, MA, USA) programing language and the motion equations were solved numerical by using the Runge-Kutta ordinary differential equation solver ode45s with an adaptive time-stepping scheme. A maximum integration step time of 10^−4^ s was defined to ensure numerical stability of solution at every time step. The absolute and relative tolerance of the solver was set to 10^−6^. These parameters yielded stable and reliable results of the analyzed problem at a reasonable simulation time.

**Figure 3:**
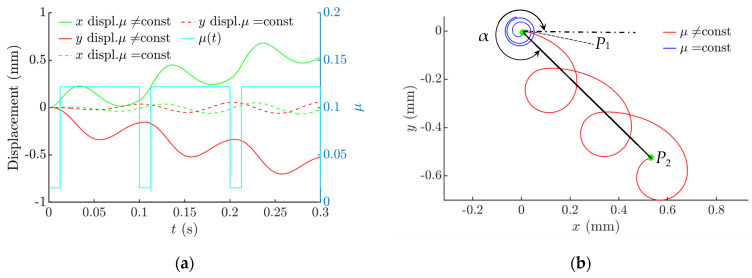
Displacement of the part when *µ*_1_ = 0.122, *µ*_2_ = *µ*_1_/8, *g* = 9.81 m/s^2^, *A_e_* = 0.31 mm, *ω* = 62.8 rad/s, *ε = π*/2, *ϕ* = 0, Δ*τ* = *π*/4: (**a**) displacement vs. time and the effective dry friction coefficient vs. time; (**b**) motion trajectory of the part and the angle of displacement *α*.

**Figure 4:**
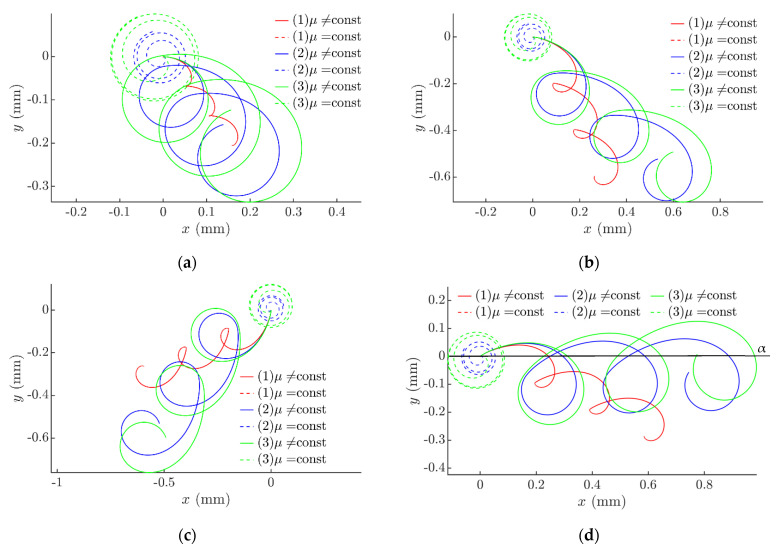
Trajectories of the part when *µ*_1_ = 0.122, *µ*_2_ = *µ*_1_/8, *g* = 9.81 m/s^2^, *ω* = 62.8 rad/s, *ε = π*/2; (1) *A_e_* = 0.28 mm, (2) *A_e_* = 0.31 mm, and (3) *A_e_* = 0.32 mm; (**a**) *ϕ* = 0, Δ*τ* = *π*/10; (**b**) *ϕ* = 0, Δ*τ* = *π*/4; (**c**) *ϕ* = 11*π*/9, Δ*τ* = *π*/4; and (**d**) *ϕ* = 17*π*/9, Δ*τ* = *π*/4.

When the platform circularly oscillates in the horizontal plane, and the dry friction is constant, the part can be moved at some distance initially in a spiral or wavy trajectory, depending on the excitation parameters. However, eventually the part starts to oscillate around the point of equilibrium in a circular trajectory (Figure 3, as *µ* = const) [41]. To make the part move along a prescribed direction, it is necessary to create a system asymmetry. In this research, a frictional system asymmetry is proposed when the effective coefficient of dry friction is being dynamically reduced for some fraction of the period of the circular platform’s oscillations. In this case, the part continues to be moving (Figure 3, as *µ* ≠ const). This sort of dynamic dry friction control allows one to control the motion of the part as well. The nature of the part’s motion depends on the dry friction control parameters (Figure 4). Therefore, this technique can be applied for conveying and nonprehensile manipulation tasks, as parts can be displaced at any distance and in any direction. The trajectories of the part after 3 circular oscillation cycles are presented in Figure 3.

In this research, the covered distance was considered to be the strait line connecting the starting point *P*_1_ and the ending point *P*_2_ of the trajectory (Figure 3b). The displacement angle α is considered to be an angle between the horizontal and the covered distance (Figure 3b).

The modeling of the manipulation process with dry friction control showed that both the direction and the velocity of the motion of the part can be controlled over a wide range by changing *ϕ* and Δ*τ*. It was found that the nature of the trajectory of the part’s motion mainly depends on Δ*τ* (Figure 4a,b). When Δ*τ* increases, a higher system asymmetry is created, and due this reason the displacement of the part increases at the same number of circular oscillation cycles, since the trajectory becomes less circular (more stretched).

The angle of displacement mainly depends on the phase shift *ϕ* (Figure 4c,d). The angle of the net force vector affecting the part per cycle is influenced by *ϕ* since the function for dynamic dry friction control that causes the frictional system asymmetry is shifted by *ϕ* from the function defining the circular motion of the platform.

**Figure 5:**
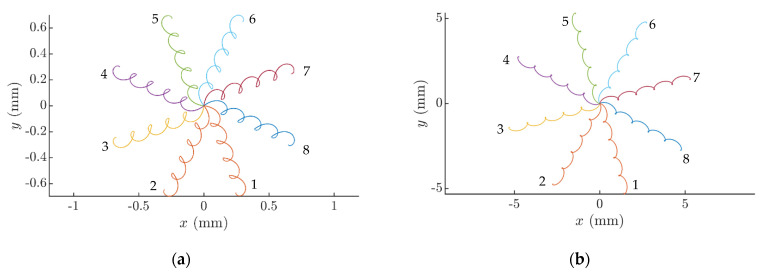
Trajectories of the part at different values of the phase shift *ϕ* when *µ*_1_ = 0.12, *µ*_2_ = *µ*_1_/8, *g* = 9.81 m/s^2^, *A_e_* = 0.28 mm, *ω* = 62.8 rad/s, *ε = π*/2, (1), *ϕ* = 0, (2), *ϕ* = *π/*4; (3), *ϕ* = *π/*2; (4)*, ϕ* = 3*π/*4; (5)*, ϕ* = *π*; (6)*, ϕ* = 5*π/*4; (7), *ϕ* = 3*π/*2; (8), *ϕ* = 7*π/*4: (**a**) as Δ*τ* = *π*/6; (**b**) as Δ*τ* = *π*.

The angle of displacement *α* can be controlled in a full range from 0 to 2*π* by changing the phase shift *ϕ* (Figure 5). Figure 5 also demonstrates how the nature of the trajectory is being controlled by changing Δ*τ*. At a lower Δ*τ* value (Δ*τ* = *π*/6), the displacements are significantly lower, and the trajectories are more circular (Figure 5a) compared to the trajectories with a higher Δ*τ* (Δ*τ* = *π*) presented in Figure 5b. Higher levels of the system asymmetry create higher net forces affecting the part per cycle, resulting in a displacement. Due to this and since Δ*τ* is associated with the level of the asymmetry of the analyzed system, the trajectories at the higher value of Δ*τ* are less stretched and the displacements are significantly higher in Figure 5b.

**Figure 6:**
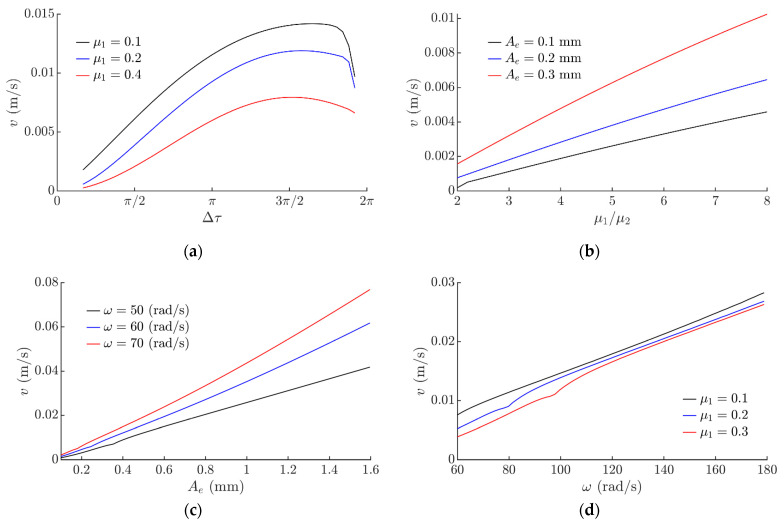
Mean displacement velocity depending on: (**a**) Δ*τ* when *µ*_2_ = *µ*_1_/8, *g* = 9.81 m/s^2^, *A_e_* = 0.28 mm, *ω* = 62.8 rad/s, *ε = π*/2, *ϕ* = 0; (**b**) *µ*_1_/*µ*_2_ when *µ*_1_ = 0.1, *g* = 9.81 m/s^2^, *ω* = 62.8 rad/s, *ε =* π/2, *ϕ* = 0, Δ*τ* = *π*/6; (**c**) circular excitation amplitude *A_e_* when *µ*_1_ = 0.1, *µ*_2_ = *µ*_1_/8, *g* = 9.81 m/s^2^, *ε = π*/2, *ϕ* = *π*/2, Δ*τ* = 2*π*/3; (**d**) circular excitation frequency *ω* when *µ*_2_ = *µ*_1_/8, *g* = 9.81 m/s^2^, *ε = π*/2, *A_e_* = 0.28 mm, *ϕ* = *π*/2, Δ*τ* = 2*π*/3.

The influence of the system parameters on the mean displacement velocity *v* of the part was determined. The mean displacement velocity *v* was calculated by dividing the covered distance after 20 circular oscillation cycles by the traveling time. As Δ*τ* increases up to 3*π/*2, the mean displacement velocity *v* also increases (Figure 6a). This is attributed to a higher asymmetry of the system. However, a further increase in Δ*τ* results in a decrease in *v* (Figure 6a), since the system asymmetry starts to decrease with a further increase in Δ*τ* in this range.

Figure 6b shows how *v* depends on the ratio of the nominal dry friction coefficient *µ*_1_ to the reduced effective dry friction coefficient *µ*_2_. The ratio *µ*_1_/*µ*_2_ has a significant influence on the mean displacement velocity *v*. As *µ*_1_/*µ*_2_ increases, the displacement velocity *v* increases as well (Figure 6b). This is also attributed to a higher asymmetry of the system as higher *µ*_1_/*µ*_2_ ratios results in a higher level of the system asymmetry. The mean displacement velocity *v* increases as *A_e_* increases (Figure 6c). Similarly, as *ω* increases, *v* increases as well (Figure 6d) due to a higher angular velocity of the platform. Also, higher *v* values were observed at lower *µ*_1_ values (Figure 6b,d). The modeling revealed that *v* does not depend on the phase shift *ϕ*.

**Figure 7:**
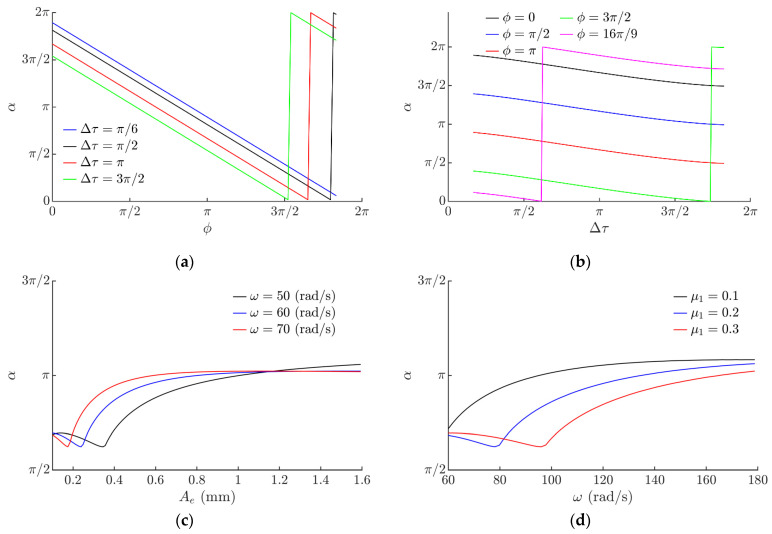
The displacement angle *α* depending on: (**a**) phase shift *ϕ* when *µ*_1_ = 0.1, *µ*_2_ = *µ*_1_/8, *g* = 9.81 m/s^2^, *A_e_* = 0.28 mm, *ω* = 62.8 rad/s, *ε = π*/2; (**b**) Δ*τ* when *µ*_2_ = *µ*_1_/8, *g* = 9.81 m/s^2^, *A_e_* = 0.28 mm, *ω* = 62.8 rad/s, *ε =* π/2; (**c**) excitation amplitude *A_e_* when *µ*_1_ = 0.1, *µ*_2_ = *µ*_1_/8, *g* = 9.81 m/s^2^, *ε =π*/2, *ϕ* = *π*/2, Δ*τ* = 2*π*/3; (**d**) excitation frequency *ω* when *µ*_2_ = *µ*_1_/8, *g* = 9.81 m/s^2^, *ε = π*/2, *A_e_* = 0.28 mm, *ϕ* = π/2, Δ*τ* = 2*π*/3.

The modeling showed that the displacement angle *α* varies in a full range from 0 to 2*π* depending on the phase shift *ϕ*. A linear relation between *α* and *ϕ* was observed (Figure 7a). It was demonstrated that Δ*τ* has some influence on the displacement angle α (Figure 7b). As Δ*τ* increases, α slowly decreases. Sudden changes in α depicted in Figure 7a,b is due to the fact that the zero value of the angle (the horizontal) is crossed (Figure 4d). The ratio *µ*_1_/*µ*_2_ does not have a high influence on the displacement angle α. Figure 7c shows the influence of the circular excitation amplitude on the displacement angle *α*. At higher amplitudes (higher than 1 mm), a negligible influence of *A_e_* on the displacement angle α was observed (Figure 7c). Similar processes were observed as the angular frequency of the circular excitation was varying (Figure 7d). The modeling demonstrated that the ratio *µ*_1_/*µ*_2_ does not have a high influence on the displacement angle α.

## 3. Experimental Analysis

### 3.1. Methodology

**Figure 8:**
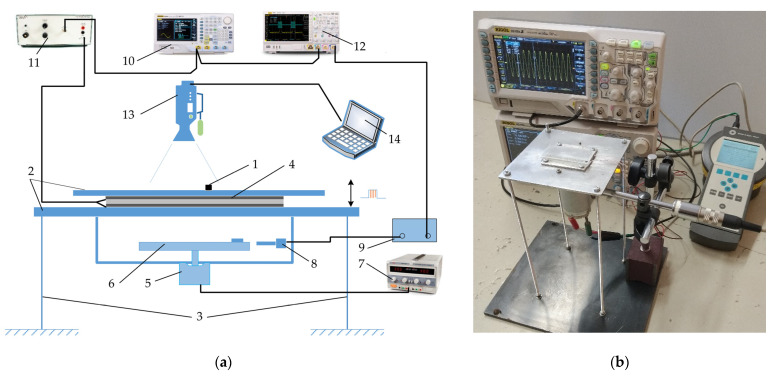
Experimental setup for manipulation of parts on a horizontal circularly oscillating platform with dynamic dry friction control: (**a**) scheme where (1) part to be manipulated; (2) platform; (3) elastic rods; (4) piezoelectric actuator; (5) electric motor; (6) eccentric mechanism; (7) electric motor power supply; (8) optical reference sensor, (9) vibration analyzer; (10) arbitrary waveform generator; (11) high frequency vibration amplifier; (12) digital oscilloscope; (13) video camera; (14) computer; (**b**) general view of the platform for nonprehensile manipulation with dry friction control.

An experimental analysis of manipulation of parts on a horizontal circularly oscillating platform with dynamic dry friction control was carried out to verify the theoretical findings. To control the effective dry friction dynamically in respect of the period of the circular excitation, high frequency vibrations were applied. The scheme of the mechatronic system used for the experiments is presented in Figure 8a, and a general view of the experimental platform for nonprehensile manipulation with dry friction control is presented in Figure 8b.

The setup consists of a part (1) to be manipulated which is placed on a platform (2) mounted on four elastic rods (3) (Figure 8a). The upper part of the platform is made of duralumin, and its upper surface is polished to an average surface roughness of about 0.4 μm. A piezoelectric actuator (4) is mounted between the upper and lower parts of the platform. An electric motor (5) with an eccentric mechanism (6) is attached to the lower part of the platform. The electric motor is powered by a direct current power supply (7) (HY3002-2, Mastech, Shenzhen, China). Circular oscillations of the platform are excited by the eccentric mechanism. 

The phase of the circular excitation is monitored by an optical reference sensor (8) (P–95, Brüel and Kjær). The signal of the optical reference sensor is processed by a vibration analyzer (9) (Vibrotest 60, Brüel and Kjær, Nærum, Denmark). The signal of high-frequency excitation for the piezoelectric actuator is generated by an arbitrary waveform generator (11) (DG4202, RIGOL, Beijing, China) and amplified by a piezo linear amplifier (11) (EPA-104, Piezo Systems Inc., Cambridge, MA, USA). The signal for the piezoelectric actuator is composed of high frequency pulse sequences, which are synchronized in respect to the phase of the circular excitation. Similarly, like it is shown in Figure 2, the piezoelectric actuator is activated for a fraction of Δ*τ* in each period of the circular excitation of the platform with a phase shift of *ϕ*. The frequency of the high-frequency excitation for the piezoelectric actuator was 5890 Hz. The piezoelectric actuator was periodically exciting vertical vibrations of the platform’s upper surface with an amplitude of 0.5 µm. When the piezoelectric actuator is activated, the effective friction force is reduced as a result of the dynamic processes, which occur in the contact between the part and the platform. Employing this technique, the effective dry friction force between the part and the platform can be dynamically controlled in a predefined manner. The output from the optical reference sensor as well as the signal for the piezoelectric actuator generator are monitored by a digital oscilloscope (12) (DS1054, RIGOL).

A high-speed camera (13) (Phantom v711, 1280 × 800 CMOS sensor, 1 Mpx, 20 µm pixel size) was used to record the displacement of the part. The camera was equipped with a Canon MP-E 65 mm f/2.8 1-5x Macro Lens. The camera was controlled by a computer (14). To digitize the displacement of the part, a video processing program was developed, employing the normalized cross-correlation approach.

### 3.2. Experimental Results

Using the mechatronic system shown in Figure 8, experiments of the proposed method employing dynamic dry friction control were carried out manipulating a cylindrical silver-coated part (*ϕ*6 × 0.8 mm, 0.201 g).

**Figure 9:**
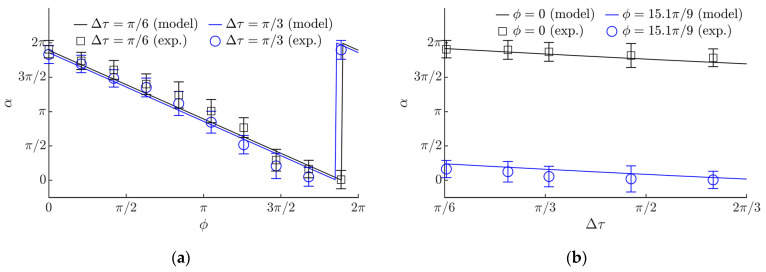
Experimental and modeling results of *α* depending on: (**a**) the phase shift *ϕ* when *µ*_1_ = 0.1, *µ*_2_ = *µ*_1_/8, *g* = 9.81 m/s^2^, *A_e_* = 0.28 mm, *ω* = 62.8 rad/s, *ε = π*/2; (**b**) Δ*τ* when *µ*_1_ = 0.1, *µ*_2_ = *µ*_1_/8, *g* = 9.81 m/s^2^, *A_e_* = 0.28 mm, *ω* = 62.8 rad/s, *ε = π*/2.

The experiments verified the theoretical findings and demonstrated that the angle of displacement *α* can be controlled by changing the phase shift *ϕ* (Figure 9a). A comparison between the modeling and experimental results was made. The dependency of *α* on *ϕ* obtained by the model showed a good agreement with the experimental values (Figure 9a). Figure 9b shows a comparison of the experimental and modeling results of the displacement angle *α* depending on Δ*τ*. The experimental results confirmed that Δ*τ* has a less significant influence on *α* (Figure 9b). The modeled dependence of *α* depending on Δ*τ* also showed a good agreement with the experimental values.

**Figure 10:**
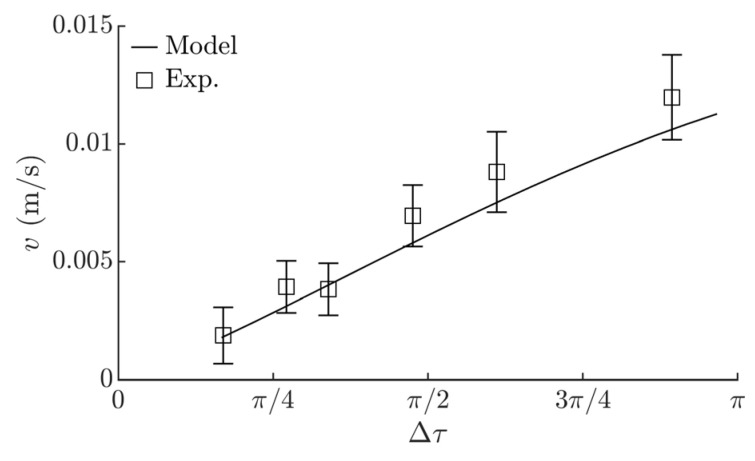
Experimental and modeling results of the mean displacement velocity depending on Δ*τ* when *µ*_1_ = 0.1, *µ*_2_ = *µ*_1_/8, *g* = 9.81 m/s^2^, *A_e_* = 0.28 mm, *ω* = 62.8 rad/s, *ε = π*/2, *ϕ* = 0.

Experimental and modeling results of *v* vs. Δ*τ* are shown in Figure 10. In the analyzed range, an increase in the mean displacement velocity *v* was observed as Δ*τ* was increasing. A comparison between the modeling and the experiments also showed a good agreement. This confirms the theoretical findings and demonstrates how the mean displacement velocity depends on the level of the system asymmetry. 

**Figure 11:**
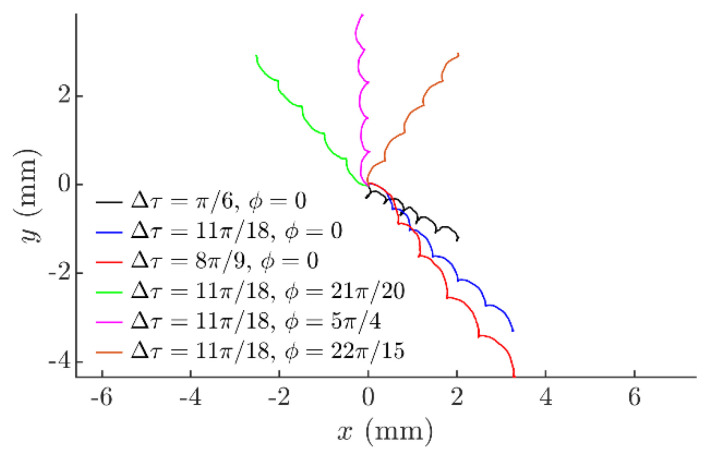
Captured experimental trajectories of the part when *A_e_* = 0.28 mm, *ω* = 62.8 rad/s, *ε = π*/2.

Figure 11 shows part’s trajectories at various *ϕ* and Δ*τ* values captured during the experiments. The trajectories demonstrate how the part is directed towards any direction by changing the phase shift *ϕ*. Figure 11 also demonstrates that the part is displaced further under higher Δ*τ* values, after the same number of circular oscillation cycles. This nature of the part’s motion was close to the one observed in the modeling results.

## 4. Conclusions

A novel method of nonprehensile manipulation of parts on a horizontal platform oscillating in circular motion with dynamic friction control is proposed.

A mathematical model of the proposed manipulation method was developed and solved numerically to provide a theoretical verification of the functionality of the proposed method as well as determine the control parameters that define the motion characteristics. The modeling results showed that by changing the phase shift *ϕ* between the function for dynamic dry friction control and the function defining the circular motion of the platform, the part can be moved in any direction as the angle of displacement can be controlled in a full range from 0 to 2*π*. The nature of the trajectory of the part’s motion mainly depends on the width Δ*τ* of the rectangular function for dynamic dry friction control. This parameter can be associated to the level of the system asymmetry. When Δ*τ* increases, the displacement of the part increases at the same number of circular oscillation cycles, since the motion trajectory of the part becomes less circular (more stretched). Also, the mean displacement velocity *v* increases as Δ*τ* increases. This is attributed to a higher asymmetry of the system. However, when a critical value of Δ*τ* is exceeded, *v* starts to decrease since the system asymmetry becomes lower again.

To verify the theoretical findings, an experimental setup for manipulation of parts on a horizontal circularly oscillating platform with dynamic dry friction control was developed and manufactured. The experimental results qualitatively confirmed the theoretical findings as the dependencies of *α* on *ϕ*, *α* on Δ*τ*, and *v* on Δ*τ* obtained by the model showed a good agreement with the experimental values. The experimentally captured nature of the part’s motion was close to the one observed in the modeling results.

The research results demonstrate that the proposed method of creating a frictional system asymmetry by employing dynamic dry friction control can be applied for conveying and omnidirectional nonprehensile manipulation tasks. As the technical equipment for the implementation of this method is relatively simple and compact, from the practical point of view, this method is very versatile and can be used in various environments with objects/parts of various sizes.

The presented analysis enriches the classical theories of nonprehensile manipulation on oscillating platforms, and the presented findings are relevant for mechatronics, robotics, mechanics, electronics, medical, and other industries.
